# Cell line access to revolutionize the biosimilars market

**DOI:** 10.12688/f1000research.14808.1

**Published:** 2018-05-03

**Authors:** Dzintars Gotham

**Affiliations:** 1 Independent Researcher, Boston, MA, USA

**Keywords:** biologics, biosimilars, economics, pharmacology, access to medicines, regulatory policy, pharmaceutical policy, health policy

## Abstract

Biologic drugs are notoriously expensive. Biosimilars, though priced lower, are also costly. Analysis of the cost of production of biologics suggests that the cost of manufacture is in many cases less than 10% of the price in high-income countries, and less than a third of the price of biosimilars in India. This in turn implies that the relatively high prices of biosimilars are largely due to the need to undertake laborious reverse-engineering and phase 3 trials to demonstrate clinical similarity. In this article, it is proposed that originators could be required to submit cell line stocks to regulators and disclose details of manufacturing processes. These would be shared with prospective non-originator manufacturers to greatly reduce the investments needed to bring a non-originator biologic to market. This system would allow far greater price reductions for biologics after the expiry of monopoly rights (e.g. patents), while maintaining the monopoly rights used to incentivize drug development.

## Introduction

The importance of biologics in both clinical and economic terms continues to grow ^1^. At the same time, biologics are notoriously expensive, and challenges in affordability remain even after the expiry of monopoly rights. Price reductions seen with biosimilars are in general smaller than for small molecule medicines and have a higher starting point, with originator prices typically being in the tens of thousands of dollars. Even after originator patent expiry, biosimilars strain health budgets and remain inaccessible for many patients globally ^2, 3^.

## Biologic monopoly rights and biosimilar development

Biologic drugs are large, complex molecules whose exact composition is often not fully known, and the exact manufacturing process used is closely related to safety and efficacy – including the specific growth media, post-translational modifications, and the purification processes used. Details on these manufacturing processes are trade secrets and are thus inaccessible to competitors for a potentially indefinite period. Moreover, the cell line that produces the originator product remains the property of the originator like any other physical asset, and almost all information about the cell line remains a trade secret. Consequently, biosimilar manufacturers have to undertake laborious reverse-engineering work and phase 3 trials to demonstrate that their product is not different to the originator’s in a clinically significant way.

This represents a significant duplication of efforts and means that the barriers to competition in biologics far exceed the time-limited monopoly rights – granted through patents, market exclusivity, and data exclusivity – that attempt to legislatively balance the need to incentivize drug development against the social benefit of competition-driven price reductions. These barriers translate to higher costs: While the exact numbers are not publicly available, the cost of bringing a biosimilar to market in the US has been estimated to be $100–200 million, compared to $1–5 million for small-molecule medicines ^4^.

Aside from development expenditures, what are the inherent costs of manufacturing a biosimilar once it has been approved? For monoclonal antibodies – the most common type of biologic drug – they are reported to range US$20,000–300,000 per kilogram of active ingredient, which includes capital investments in manufacturing facilities and overheads ^5, 6^. Multiplying these estimates by per-treatment dosage, the costs of manufacturing alone appear to represent only a small proportion of the price – 0.001–6% of current lowest prices in the US and 0.004–14% of prices in the UK, for blockbuster biologics ( Table 1). Even in India, where the costs of bringing a biosimilar to market are considered to be significantly lower, and all the prices shown are for biosimilars, the estimated cost of manufacturing would represent only 0.02–27% of the price.

**Table 1.** Current prices of selected blockbuster biologics and cost of manufacture for the active ingredient. The table illustrates the large differences between current prices and the cost of manufacturing the active ingredient, for blockbuster biologic medicines.

Medicine	Example indication	Duration of treatment used for comparison (corresponding dosage in mg)	Lowest available price (USD)			Cost of manufacturing the active ingredient
			US (Veterans Affairs)	UK	India	
Adalimumab	Rheumatoid arthritis	2-week cycle (40mg)	$707	$482	$385 *	$1–12
Alemtuzumab	Relapsing- remitting multiple sclerosis	2-year treatment course (96mg)	$122,477	$77,213	N	$2–29
Bevacizumab	Metastastic colorectal cancer	2-week cycle (700mg)	$3,694	$2,216	$1,077 *	$14–210
Etanercept	Rheumatoid arthritis	1 month of treatment (200mg)	$2,019	$3,526 *	$639 *	$4–60
Infliximab	Crohn’s disease	1 maintenance dose (350mg)	$1,753 *	$1,808 *	$1,723 *	$7–105
Ranibizumab	Wet age- related macular degeneration	1 intravitreal injection (0.5mg)	$1,300	$229	$57 *	$0.01–0.15
Rituximab	Non-Hodgkin’s lymphoma	1 cycle (650mg)	$3,685	$1,400 *	$711 *	$13–195
Trastuzumab	HER2-positive breast cancer	3-week cycle (420mg)	$2,878	$1,172	$861 *	$8–126

*Biosimilar. Details on indications, dosage, and price sources are available in Supplementary File 1. Prices for the UK may not include confidential rebates.

It thus appears that if the need for investments in reverse-engineering and phase 3 trials were removed, price reductions for biologic therapies following the expiry of monopoly rights could be far greater than at present.

## A proposal: cell line access

In order to lower the barriers to bringing follow-on biologic products to market, prospective manufacturers should be given access to the originator cell lines and detailed descriptions of manufacturing processes.

To achieve this, originator biologics manufacturers could be required to submit a living vial of the cell line used to manufacture the product upon regulatory approval, to be stored by the relevant regulatory body. Cloned cell lines would then be shared with prospective manufacturers, prior to the expiry of the originator’s monopoly rights. Detailed descriptions of originators’ manufacturing processes, which are already submitted to regulators confidentially, could be made publicly available ^7^.

This system would allow originators to fully enjoy legislatively granted time-limited monopoly rights, while avoiding arguably unnecessary and duplicative drug development and enabling maximal competition as soon as monopoly rights expire.

I refer to this proposed system as ‘cell line access’ and biologics developed through this system as CLA biologics. The non-profit group Knowledge Ecology International has previously made a similar proposal ^8^, and Price and Rai have proposed that incentive mechanisms could be set up to encourage the disclosure of trade secrets pertaining to manufacturing processes ^7^.

## The benefits of cell line access

First, access to cell lines and production processes would dramatically reduce the financial and time investments required for developing a non-originator biologic product. The prices of CLA biologics would be expected to be substantially lower than the price reductions currently seen for biosimilars, both due to increased competition and lower costs.

Second, there would be clinical benefits, as CLA biologics could be expected to automatically be interchangeable with the originator product, rather than only ‘similar’.

Third, biopharmaceutical science more broadly would benefit, especially if access to cell lines and manufacturing processes were extended also to research institutions.

## Adapting existing regulatory procedures

The potential benefits of cell line access would in large part depend on regulators being satisfied that if an identical cell line and manufacturing process are used, phase 3 trials would not be necessary, and approval could be granted based on demonstration of equivalence by other methods.

Differences between CLA biologics and the reference (originator) product would be expected to be of the same magnitude as batch-to-batch variation in the originator’s product. As originators regularly make slight changes to their manufacturing process, regulatory procedures are already in place to ensure that these changes do not jeopardize efficacy or safety ^9^, for which *in vitro* analytical tools are nearly always sufficient ^9, 10^. These established procedures could be adapted to the task of ensuring that CLA biologics are not substantially different from the originator product.

Similarly, the change of manufacturer, while preserving the cell line and manufacturing processes used, is comparable to situations where originator companies use contract manufacturing organizations or regional manufacturing partners, an increasingly common practice ^11^.

## Conclusion

Enabling prospective non-originator manufacturers to access originator cell lines and manufacturing processes could remove the need for laborious reverse-engineering and duplicative clinical trials. This would allow full enjoyment by originators of the monopoly rights granted through patents and other protections, while allowing far greater price reductions upon expiry of monopoly rights. While significant political challenges would be expected, this proposed mechanism could be explored further in terms of the potential economic and health impacts, and practical options for implementation.

## Data availability

No data is associated with this article.

## Supplementary material

Supplementary File 1. This document describes the data sources for the US, UK, and India prices given in Table 1, as well as assumptions regarding dosage.
