# Supplementary material for: Cell line access to revolutionize the biosimilars market
Source: F1000Res. 2018 May 3;7:537. [Version 1] doi: 10.12688/f1000research.14808.1 (PMC6051195; doi:10.12688/f1000research.14808.1)
Supplement: Supplementary file 1 [file f1000research-7-16117-s0000.tgz › 25f6c1b2-0164-4878-8370-b2b52b1e4534.docx]

**Supplementary information**

**Indications and dosage**

Indications, information of treatment cycles, and dosing information were gathered from the electronic Medicines Compendium (eMC), which provides product information from the European Medicines Agency (EMA) and the UK Medicines and Healthcare Products Regulatory Agency (MHRA).^1^

Trastuzumab: 6mg/kg maintenance dose and 70kg body weight assumed.

Bevacizumab: 10mg/kg dose and 70kg body weight assumed.

Infliximab: 5mg/kg dose and 70kg body weight assumed.

Rituximab: 375mg/m^2^ dose and 1.73m^2^ body surface area assumed.

For all prices, perfect vial sharing was assumed.

**Calculation of cost of producing the active ingredient**

The cost of producing the active ingredient was calculated by multiplying the amount, in milligrams, of active ingredient needed over the treatment duration (i.e. the dosage) with the estimates reported by Kelley of $20,000 to $300,000 per kilogram of active ingredient, equivalent to $0.02–0.3 per milligram.^2^

**Price sources**

Prices for the US are from the US Department of Veterans Affairs (VA) National Acquisition Center Pharmaceutical Catalog Search.^3^ VA prices, rather than other prices, such as the National Average Drug Acquisition Cost (NADAC), were used to make the comparison conservative, as VA prices are in general some of the lowest prices available in the US.^4^

Three different prices are listed in this catalog: the Federal Supply Schedule price, the VA National Contract price, and the Big 4 price. The lowest price across all three, and across dosage forms, was selected.

For the UK, the lowest price reported in the British National Formulary was selected. Other public price data sources, such as the Prescription Cost Analysis and drugs and pharmaceutical electronic market information tool (eMIT) were reviewed, but did not contain data on the medicines compared.^5,6^

For India, the lowest available price was selected from a private market price comparison website.^7^

**Exchange rates**

Exchange rates were collected from [www.xe.com](http://www.xe.com) on the March 1, 2018:

1 GBP = 1.37 USD

1 INR = 0.015 USD

**References to the supplementary file**

1 Datapharm. electronic Medicines Compendium. [https://www.medicines.org.uk/emc/](https://www.medicines.org.uk/emc/%20) (accessed March 25, 2018).

2 Kelley B. Industrialization of mAb production technology: The bioprocessing industry at a crossroads. *mAbs* 2009; **1**: 440–449.

3 U.S. Department of Veterans Affairs. Pharmaceutical Catalog Search. <https://www.va.gov/nac/Pharma/List> (accessed March 25, 2018).

4 Congressional Budget Office. Prices for Brand-Name Drugs Under Selected Federal Programs. 2005. <http://www.cbo.gov/sites/default/files/cbofiles/ftpdocs/64xx/doc6481/06-16-prescriptdrug.pdf> (accessed April 2, 2018).

5 NHS Digital. Prescription Cost Analysis, England. <https://data.gov.uk/dataset/prescription-cost-analysis-england> (accessed April 2, 2018).

6 Department of Health. Drugs and pharmaceutical electronic market information tool (eMit). <https://www.gov.uk/government/publications/drugs-and-pharmaceutical-electronic-market-information-emit> (accessed April 2, 2018).

7 1mg. <http://www.1mg.com/> (accessed April 2, 2018).
