# Supplementary material for: Cell line access to revolutionize the biosimilars market
Source: F1000Res. 2018 May 3;7:537. [Version 1] doi: 10.12688/f1000research.14808.1 (PMC6051195; doi:10.12688/f1000research.14808.1)
Supplement: Peer review history

# Peer review: Referee response for version 1

**DOI:** 10.5256/f1000research.16117.r34194

**Published:** 2018-07-12

**Authors:** François Bocquet

**Affiliations:** 1 Pharmacy Department, General Agency of Equipments & Health Products, University Public Hospitals of Paris, Paris, France

This opinion paper is interesting. My first concern is the presentation of the production costs of the active substances.

 Indeed, only reasoning (ie for each molecule) in proportion to the Kelley figure of $20,000 to $300,000 per kilogram of active ingredient (equivalent to $ 0.02-0.3 per milligram) without having any idea of the real cost for each molecule can be misleading, and is not very satisfactory. These data being confidential, thank you to the authors to specify this in the legend of the table and not only in the supplementary material.

 Even if the idea proposed by the authors is interesting intellectually, it clashes with the respect of the patent right. It should be emphasized more in the paper.

I have read this submission. I believe that I have an appropriate level of expertise to confirm that it is of an acceptable scientific standard.

## References

[1] BocquetFLoubièreAFusierICordonnierALPaubelP: Competition Between Biosimilars and Patented Biologics: Learning from European and Japanese Experience. *Pharmacoeconomics*.2016;34(11) : 10.1007/s40273-016-0428-6 1173-1186 10.1007/s40273-016-0428-6 27412251

# Peer review: Referee response for version 1

**DOI:** 10.5256/f1000research.16117.r34142

**Published:** 2018-05-31

**Authors:** Amit Misra

**Affiliations:** 1 CSIR- Central Drug Research Institute (CDRI), Lucknow, Uttar Pradesh, India

My compliments to the author for this potentially revolutionary idea. However, solely addressing the drug regulator and enjoining it to implement cell line access (CLA) is, in my view, a sub-optimal strategy. First, the regulator may very often cite lack of capacity to preserve and distribute vials containing live cells. Issues related to refusal of access (e.g., on grounds of lack of competence or non-compliance with Good Manufacturing Practices) to applicants would require the regulator to expend a great deal of time and effort in deciding who gets a vial and who doesn't. Last, the innovator is denied any say in the dissemination of the innovation for public good if the drug regulator is the sole arbiter of CLA. With competitive marketing practices being what they are, it is not inconceivable that a competitor may choose to acquire CLA, only to generate a sub-optimal product that could then be faulted for poor performance to build a negative perception about the innovator.

 In my view, it would be important to invoke a "multi-stakeholder initiative" (MSI) involving drug regulators, civil society organizations working on access to medicines, and corporations that discover and produce biologics to ensure CLA worldwide. "Corporate Social Responsibility" (CSR) guidance and legislation in various countries would provide incentive to corporations to join such an MSI. A transparent process whereby applicants requesting access could be assessed and granted (or refused) CLA would be, in my view, a more sustainable strategy. Originators could then also "hand-hold" successful applicants through the process of adapting the cell lines to their specific bioreactors and downstream processing equipment. Of course, drug regulators would be required to notify CLA as mandatory after the monopoly period is over, while granting marketing permission (as also retrospectively for off-patent biologics).

 I would like to request the author to consider the feasibility and sustainability of the CLA initiative before concluding that it is sufficient for the drug regulator to require originators to share cell lines and manufacturing processes as a "practical option for implementation."

 Finally, although it is beyond the scope of the present article, I would like to suggest that the author may touch upon the regulatory requirement of "sameness" in respect of biologics, and whether Phase 3 "comparative" clinical trials actually make sense. There is extensive literature on these aspects, including two articles cited below.

I have read this submission. I believe that I have an appropriate level of expertise to confirm that it is of an acceptable scientific standard.

## References

[1] MinghettiPRoccoPSchellekensH: The constrained prescription, interchangeability and substitution of biosimilars. *Nature Biotechnology*.2015;33(7) : 10.1038/nbt.3272 688-689 10.1038/nbt.3272 26154003

[2] SchellekensHSmolenJDicatoMRifkinR: Safety and efficacy of biosimilars in oncology. *The Lancet Oncology*.2016;17(11) : 10.1016/S1470-2045(16)30374-6 e502-e509 10.1016/S1470-2045(16)30374-6 27819248
